# The Impact of Sleep Dysfunction on Inflammatory Skin Diseases: A Systematic Review

**DOI:** 10.7759/cureus.99598

**Published:** 2025-12-19

**Authors:** Tahani A Moafa, Mohammed E Mojiri, Khlood K Alattas, Ali M Sumayli, Esra H Alnujaidi, Maha O Alharbi, Ramis M Manni, Nawal H Hibili, Faris A Alomir, Faris M Almutairi, Saleh M Alhusayni

**Affiliations:** 1 Family Medicine, Jazan Health Cluster, Jazan, SAU; 2 Medicine, Jazan University, Jazan, SAU; 3 Medicine, King Faisal University, Hofuf, SAU; 4 Medicine, Sulaiman Alrajhi University, Bukayriyah, SAU; 5 Faculty of Medicine, Jazan University, Jazan, SAU; 6 Faculty of Medicine, Umm Al-Qura University, Makkah, SAU; 7 Faculty of Medicine, King Faisal University, Hofuf, SAU; 8 Medicine, King Saud Bin Abdulaziz University for Health Sciences, Riyadh, SAU

**Keywords:** atopic dermatitis, dermatology, inflammation, nocturnal symptoms, pruritus, psoriasis, quality of life, sleep disturbance, sleep quality, systematic review

## Abstract

Sleep disturbance is increasingly recognized as a major comorbidity in chronic inflammatory skin diseases, driven by altered sleep architecture, circadian disruption, and pruritus-related nocturnal symptoms that exacerbate disease activity and reduce quality of life. This systematic review of 13 studies (N ≈ 189,000) identified the prevalence, correlates, and clinical implications of sleep disturbance across major dermatologic conditions, focusing on atopic dermatitis (AD) and psoriasis. In AD, poor sleep showed strong associations with higher Scoring Atopic Dermatitis (SCORAD) and Patient-Oriented Eczema Measure (POEM) scores and greater pruritus intensity, with itch emerging as the primary predictor of sleep disruption. In psoriasis, poor sleep was closely linked to higher Psoriasis Area and Severity Index (PASI) and Dermatology Life Quality Index (DLQI) scores and more severe pruritus, with depression mediating the impact of quality-of-life impairment on sleep. Genetic evidence further supported a causal relationship between insomnia and increased risk of AD and psoriasis. Collectively, findings reveal a bidirectional interplay in which nocturnal pruritus, emotional distress, and inflammation sustain one another, underscoring sleep disturbance as an integral component of dermatologic disease burden and highlighting the need for routine sleep assessment, targeted interventions, and longitudinal studies to determine whether improving sleep can reduce inflammatory activity and enhance patient outcomes.

## Introduction and background

Sleep is essential for maintaining homeostasis, supporting tissue repair, and regulating immune function. Disrupted sleep contributes to widespread health consequences and may directly influence skin physiology, as the skin participates in circadian regulation and functions as both a barrier organ and a neuro-immunoendocrine interface [1-3]. Altered sleep can modify inflammatory pathways and impair tissue regeneration, creating conditions that may exacerbate dermatologic disease [4,5].

Sleep disorders are highly prevalent in inflammatory skin conditions, such as atopic dermatitis (AD), psoriasis (PSO), chronic urticaria (CU), and acne [1-3,6-10]. While pruritus and pain are the most immediate causes of nocturnal awakening, psychological distress and underlying inflammatory activity also play important roles [3,4,6]. In many patients, sleep loss is not simply a consequence of skin symptoms but a factor that may intensify immune dysregulation, worsen disease activity, and diminish quality of life [1,4,7,10].

Shared neuro-immunologic pathways further reinforce the bidirectional relationship between sleep and dermatologic inflammation. Cytokines involved in cutaneous disease also regulate sleep architecture, while circadian variations in cortisol, skin temperature, and barrier function heighten nighttime pruritus [1,3,8]. These interactions create a self-perpetuating cycle in which sleep loss and inflammation mutually exacerbate one another [2,4,6,9].

Although numerous studies have examined sleep disturbance in dermatologic conditions, findings regarding its relationship with disease severity remain inconsistent [1-13]. Variability in study design, diagnostic criteria, and outcome measures limits direct comparison across conditions. This systematic review aims to synthesize current evidence on the association between sleep disturbance and dermatologic disease severity, identify common mechanisms, and highlight gaps that can inform more integrated approaches to managing both skin disease and sleep health [1-13].

## Review

Methods

Literature Search Strategy

A systematic review was conducted in accordance with established reporting guidelines. Comprehensive searches were performed in PubMed, Cochrane Library, Web of Science, and Scopus from database inception to October 2025. Search strategies combined three major concept groups using AND, while synonyms within each concept group were combined using OR. For PubMed, the search string included the following: (sleep OR sleep disorders OR sleep disturbance OR sleep quality OR insomnia) AND (dermatology OR skin disease OR atopic dermatitis OR eczema OR psoriasis OR chronic urticaria OR acne) AND (SCORAD OR PASI OR DLQI OR “disease severity” OR severity index). MeSH terms and free-text keywords were used in parallel to maximize sensitivity. Search strategies were adapted for each database and limited to English-language publications. Two reviewers independently screened all titles and abstracts, followed by full-text review, with disagreements resolved by a third reviewer. The screening process, including reasons for exclusion, was documented in a Preferred Reporting Items for Systematic Reviews and Meta-Analyses (PRISMA) flow diagram [14]. The PubMed search strategy included terms for sleep (e.g., sleep disorders, sleep quality, insomnia), dermatology (e.g., skin diseases, AD, PSO, CU, acne), and severity indices such as the Scoring Atopic Dermatitis (SCORAD) index [15], the Psoriasis Area and Severity Index (PASI) [16], and the Dermatology Life Quality Index (DLQI) [17]. Search strategies were adapted for each database and limited to English-language publications.

Eligibility Criteria

Studies were eligible if they (1) included patients with any dermatologic condition, (2) assessed sleep disturbance or sleep quality using standardized instruments, and (3) examined the association between sleep disturbance and disease severity using validated clinical indices such as SCORAD, PASI, or DLQI. Eligible designs included cross-sectional, case-control, cohort, and Mendelian randomization studies. Excluded studies included reviews, case reports, conference abstracts, letters, editorials, non-human studies, non-dermatologic populations, and studies lacking quantitative measures of sleep or disease severity. Only full-text articles in English were included.

Data Extraction and Quality Appraisal

Data were extracted into a standardized template by two independent reviewers. Extracted variables included study characteristics, participant demographics, dermatologic condition, sleep assessment method, disease severity measure, key findings, and statistical outcomes. Quality appraisal was performed according to study design: the Appraisal Tool for Cross-Sectional Studies (AXIS) for cross-sectional studies [18], the Newcastle-Ottawa Scale (NOS) for case-control studies [19], and the Q-Genie tool for Mendelian randomization studies [20]. Each domain was rated for risk of bias, and the overall quality of included studies ranged from moderate-high to high.

Results

Study Selection

**Figure 1:**
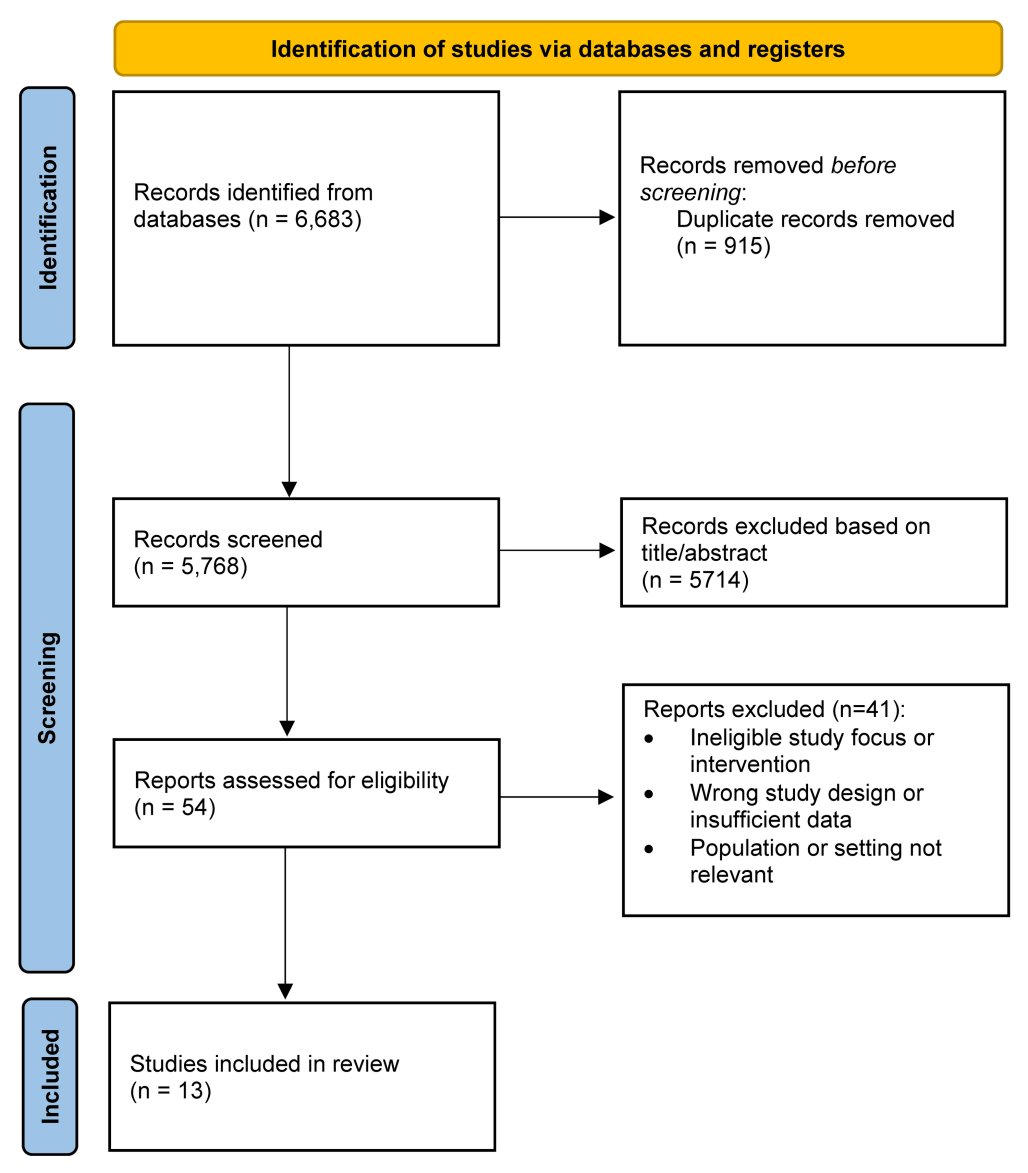
PRISMA flow diagram of the study selection process PRISMA: Preferred Reporting Items for Systematic Reviews and Meta-Analyses

The database search yielded 6,683 records. After removing duplicates, 5,768 unique studies were screened by title and abstract, resulting in the exclusion of 5,714 non-eligible records. Fifty-four full-text articles were assessed, and 41 were excluded for reasons including inappropriate study design, non-dermatologic populations, or insufficient data. Thirteen studies met all inclusion criteria and were included in the qualitative synthesis. No studies met the criteria for meta-analysis due to heterogeneity in design, populations, and outcome measures. The full screening process is presented in the PRISMA diagram (Figure 1).

Study Characteristics

The 13 included studies, published between 2015 and 2025, represented populations from Asia, Europe, North America, and Africa. Most were cross-sectional designs [2-6,9,10], with one case-control study [7] and one Mendelian randomization study [8]. Sample sizes ranged from 42 to over 180,000 participants. Age groups spanned early childhood to older adulthood, and female representation generally ranged between 40% and 60% (Table 1).

**Table 1:** Summary of the characteristics of the included studies Summary of studies evaluating sleep disturbances in dermatologic conditions. Study designs include cross-sectional surveys, clinical studies, case-control studies, and Mendelian randomization analyses. Sleep was assessed using self-reports, parent-proxy questionnaires, or validated scales, including the Pittsburgh Sleep Quality Index (PSQI), Children’s Sleep Habits Questionnaire (CSHQ), PROMIS Sleep Disturbance and Sleep-Related Impairment Scales, and Mouth Breathing Daytime/Sleep (MBD/MBS) behavioral scales. Disease severity was measured with SCORAD (SCORing Atopic Dermatitis), POEM (Patient-Oriented Eczema Measure), PASI (Psoriasis Area and Severity Index), DLQI (Dermatology Life Quality Index), PSS (Pruritus Severity Scale), and PDI (Psoriasis Disability Index). Statistical associations are reported as correlation coefficients (r), odds ratios (OR), regression coefficients (β), prevalence ratios (PR/aPR), and p-values. Adjustments were made for demographics, disease duration, comorbidities, and relevant confounders. Key findings summarize the relationships between dermatologic disease severity, sleep disturbance prevalence, and associated health outcomes. Abbreviations: AD – atopic dermatitis; DLQI – Dermatology Life Quality Index; ETS – environmental tobacco smoke; GWAS – genome-wide association study; HRQoL – health-related quality of life; ISAAC – International Study of Asthma and Allergies in Childhood; MBD – Mouth Breathing Daytime; MBS – Mouth Breathing Sleep; MR – Mendelian randomization; PASI – Psoriasis Area and Severity Index; POEM – Patient-Oriented Eczema Measure; PSQI – Pittsburgh Sleep Quality Index; PSS – Pruritus Severity Scale; PDI – Psoriasis Disability Index; SCORAD – SCORing Atopic Dermatitis; SF-36 MCS – 36-Item Short Form Mental Component Score

Study (Author)	Country	Study Design	Sample Size	Age (Mean ± SD / Range)	Sex (% Female)	Dermatologic Condition	Sleep Assessment Tool	Disease Severity Measure	Main Outcomes	Statistical Association (r / OR / β / p-value)	Adjustment Variables	Key Findings
Fishbein et al. [1]	United States	Cross-sectional national survey (PROMIS-based)	180 children with AD (5–17)	10.9 ± 3.6	44–47%	Atopic dermatitis (physician-diagnosed, stratified by severity)	PROMIS Sleep Disturbance and Sleep-Related Impairment Scales	POEM: mild, moderate, severe	Weighted prevalence of sleep disturbance: 66.9%; severe sleep disturbancee 46.1%; sleep-related impairment 61.2%	OR severe AD = 8.68 (1.82–41.49, p = 0.007); moderate AD = 2.03 (1.00–4.10, p = 0.05); itch β = 1.33 (0.62–2.04, p = 0.003); depression β = 0.67 (0.46–0.88, p < 0.01)	Adjusted for itch intensity, disease severity, age, sex, race, parental income, education	Sleep disturbance is highly prevalent (~3 million U.S. children); severity and itch independently predicted sleep disturbance; associated with depression, anxiety, fatigue, inattention, and impulsivity
Arima et al. [2]	Japan	Cross-sectional (2013 National Health and Wellness Survey, propensity-matched)	634 AD patients vs 1,268 non-AD controls	38.5 ± 12.9	0.52	Atopic dermatitis (physician-diagnosed)	Self-reported physician-diagnosed “sleep disorder” (past 12 months)	Self-rated severity (mild, moderate, severe)	Sleep disorder prevalence in AD vs non-AD and relation to severity	12.9% vs 4.6%, p < 0.001; no difference between mild and moderate/severe AD	Propensity-score matched on age, sex, marital status, income, BMI, smoking, education, insurance, and comorbidities	AD is significantly associated with higher sleep-disorder prevalence; severity is not predictive of sleep disturbance.
Ahn et al. [3]	South Korea	Cross-sectional (Korean National Health Insurance Database, 2015)	182,127 total (42,641 AD; 139,486 non-AD)	Infants to elderly (0–>65)	0.429	Atopic dermatitis (AD) compared with nonatopic eczema, urticaria, and psoriasis	ICD-10 diagnostic code G47.9 for sleep disorder	AD severity classified by treatment type: mild (topical), moderate (antihistamines), severe (systemic immunosuppressive therapy)	Prevalence and adjusted odds of sleep disorder by AD status and severity	Overall AD vs non-AD: OR = 0.85 (95% CI: 0.79–0.92, p < 0.001); mild AD OR = 0.88 (p = 0.0237); moderate AD OR = 1.31 (p = 0.0267); severe AD OR = 2.56 (p < 0.0001)	Adjusted for age, gender, economic status, AD history, and concomitant allergic diseases	Severe AD significantly increased the odds of sleep disorder; mild AD was slightly protective
Girolomoni et al. [4]	EU5 (France, Germany, Italy, Spain, UK)	Cross-sectional analysis (2017 National Health and Wellness Survey)	1,014 adults with moderate-to-severe AD	Mean 40–42	54–72%	Atopic dermatitis (self-reported, physician-diagnosed)	Self-reported insomnia or “sleep difficulties” (mild, moderate, severe)	Dermatology Life Quality Index (DLQI ≥ 6: moderate–extremely large effect)	Sleep difficulties were reported by 61.7% (moderate/severe in 37.8%); associated with poorer HRQoL and work impairment	Moderate/severe sleep difficulty linked with lower SF-36 MCS (36.4–33.1 vs 42.9, p < 0.05), lower EQ-5D (0.52–0.46 vs 0.66, p < 0.05), and higher work impairment (61.7–69.7% vs 52.3%, p < 0.05)	Controlled for age, sex, country, income, employment, BMI, comorbidities, and other atopic diseases	Over 60% of adults with moderate-to-severe AD experience sleep disturbance; severity correlated with worse QoL, work impairment, and higher healthcare utilization
Hamid & Qurtas [5]	Iraq (Erbil City, Kurdistan)	Cross-sectional clinical study	100 children (≤18)	6.84 ± 1.8 (1–18)	0.46	Atopic dermatitis (clinically diagnosed, SCORAD-based)	Sleep disturbance recorded by questionnaire (yes/no)	SCORAD index (mild 23%, moderate 69%, severe 8%)	73% reported sleep disturbance; nearly all severe AD had disturbed sleep (87.5%)	Negative correlation: SCORAD–weight centile r = –0.38, p < 0.001; SCORAD–height centile r = –0.39, p < 0.001	None reported	Sleep disturbance is prevalent in 73% of AD children; severity is inversely correlated with height and weight; poor sleep likely contributed to impaired growth
Ziyab et al. [6]	Kuwait	School-based cross-sectional epidemiological study	3,864 adolescents (11–14)	Median 12	0.561	Eczema (AD, per ISAAC criteria)	Self-reported frequency of nocturnal sleep disturbance due to an itchy rash	Current eczema and “severe eczema” (≥1 night/week sleep disturbance)	34.4% reported nocturnal sleep disturbance; 12.7% ≥1 night/week; severe eczema prevalence 1.7%	Frequent nocturnal sleep disturbance associated with cesarean delivery (aPR = 1.98, 95% CI 1.37–2.85), ETS exposure (aPR = 1.70, 95% CI 1.18–2.47), dog-keeping (aPR = 1.93, 95% CI 1.06–3.52)	Adjusted for age, mode of birth, ETS exposure, and dog-keeping	One-third of adolescents with eczema experienced nocturnal sleep disturbance; ETS and dog exposure worsened sleep; cesarean section and eczema severity increased night awakenings; antihistamine use rose with more disturbed sleep
Melikoğlu [7]	Turkey	Case-control study	58 psoriasis patients vs 58 controls	41.3 ± 12.4 (18–76)	0.45	Plaque psoriasis	Pittsburgh Sleep Quality Index (PSQI)	Psoriasis Area and Severity Index (PASI)	60.3% of psoriasis patients had poor sleep vs 10.3% of controls	Global PSQI correlated with PASI (p = 0.033); subjective sleep quality (p = 0.048), habitual sleep efficiency (p = 0.044), daytime dysfunction (p = 0.012) correlated	None reported	Psoriasis patients had worse overall and component sleep quality; disease severity was positively associated with poor sleep; sleep disturbance may aggravate psoriasis via immune-inflammatory mechanisms
Yang et al. [8]	China (Genetic data from UK Biobank & FinnGen)	Two-sample Mendelian randomization study	4 skin diseases: psoriasis (12,760), acne (4,617), AD (31,245), urticaria (13,990)	Median 24.6–51.4 (varied)	Mixed	Psoriasis, acne, AD, urticaria	GWAS-derived genetic proxies for 8 sleep traits	ICD-10 diagnostic codes for disease classification	Frequent insomnia increased the risk of psoriasis and AD; long sleep duration was protective for acne and urticaria	Psoriasis OR = 1.114 (1.011–1.227, p = 0.029); AD OR = 1.081 (1.003–1.164, p = 0.042); Acne OR = 0.972 (0.948–0.996, p = 0.029); Urticaria OR = 0.988 (0.976–1.000, p = 0.0497)	Adjusted via MR assumptions; pleiotropy tested with MR-Egger and MR-PRESSO	MR confirmed a causal link between insomnia and increased risk of psoriasis and AD; longer sleep is protective against acne and urticaria
Sahin et al. [9]	Poland	Cross-sectional clinical study with hierarchical regression and mediation analysis	42 adults with chronic plaque psoriasis	44.48 ± 17.57 (21–82)	0.405	Chronic plaque psoriasis	Pittsburgh Sleep Quality Index (PSQI)	PASI and DLQI	64.3% reported poor sleep (PSQI > 6); higher PASI, DLQI, itch intensity, and depression associated with worse sleep	PASI–PSQI r = 0.52, p < 0.01; DLQI–PSQI r = 0.68, p < 0.01; hierarchical regression: PASI β = 0.41, DLQI β = 0.37, depression β = 0.37, p < 0.01	Adjusted for age, gender, BMI, pruritus, and depression	Poorer sleep strongly correlated with disease severity and QoL impairment; depression mediated the relationship between DLQI and sleep; female sex, younger age, and higher BMI predicted worse sleep outcomes
Zaky et al. [10]	Egypt	Cross-sectional correlational clinical study	200 psoriasis patients	38.9 ± 17.8 (6–75)	0.5	Psoriasis (plaque, palmoplantar, scalp)	Pittsburgh Sleep Quality Index (Arabic version)	PASI, Pruritus Severity Scale (PSS), Psoriasis Disability Index (PDI)	Poor sleep in 16%; 50% of severe cases; PASI correlated with PSQI	PASI–PSQI p = 0.004; PSS–PSQI r = 0.687, p < 0.001; PDI–PSQI r = 0.571, p < 0.001; regression: Global sleep score = –10.09 + 0.067 × duration + 0.242 × PSS + 0.150 × PDI (R² = 0.648, p < 0.001)	Adjusted for disease duration	Poor sleep is strongly associated with higher pruritus severity and reduced QoL; disease duration, pruritus, and disability predicted sleep impairment; 50% of severe psoriasis cases are affected
Atefi et al. [11]	Iran	Cross-sectional study in dermatology clinics (Tehran, 2017)	95 children and adolescents (4–18)	Median 9 (4–18)	0.505	Atopic dermatitis (diagnosed clinically and by the 2003 National Survey of Children’s Health criteria)	Pittsburgh Sleep Quality Index (PSQI)	Not specified by score; clinical features (flexor and cheek involvement) used for disease characterization	Sleep disturbance prevalence: 32.6%; relationship with ADHD components	Sleep problem associated with hyperactivity OR = 2.91 (95% CI: 1.04–8.16, p = 0.04); attention deficit OR = 3.68 (95% CI: 1.45–9.33, p = 0.01)	Adjusted for gender and asthma status	Sleep disturbance inADHDD children predicted both hyperactivity and attention deficit; flexor involvement predicted hyperactivity, cheek involvement predicted attention deficit
Kong et al. [12]	South Korea	Cross-sectional clinical study	100 total (50 children, 50 adults)	Children: 4.9 ± 3.23; Adults: 26.4 ± 9.56	Children: 40%; Adults: 56%	Atopic dermatitis (Hanifin & Rajka criteria)	Children: CSHQ; Adults: PSQI	SCORAD index (mild <20; moderate 20–40; severe ≥40)	Children: SCORAD correlated with sleep disturbance; Adults: only certain PSQI components correlated	Children: SCORAD–CSHQ r = 0.28, p < 0.005; Adults: subjective sleep quality r = 0.387, p = 0.007; sleep latency r = 0.318, p = 0.005	None specified	Sleep quality significantly correlated with disease severity in children; in adults, specific aspects correlated; pruritus was strongly related to poor sleep; sleep problems were linked to reduced QoL
Yamaguchi et al. [13]	Japan	Population-based cross-sectional survey in nurseries	468 preschool children (2–6)	4.5 ± 1.2	0.459	Atopic dermatitis (parent-reported, physician-diagnosed)	Mouth Breathing Daytime (MBD) and Mouth Breathing Sleep (MBS) behavioral scales	No formal AD severity scale	MBD prevalence 35.5%, MBS 45.9%; AD is more prevalent among mouth breathers	MBD–AD OR = 2.4 (95% CI: 1.4–4.2, p = 0.001); MBS–AD OR = 2.4 (95% CI: 1.3–4.2, p = 0.002); adjusted MBD OR = 2.6 (1.3–5.4, p = 0.010); MBS OR = 4.1 (1.8–9.2, p = 0.001)	Adjusted for asthma, allergic rhinitis, family history (AD, asthma, rhinitis), and nasal congestion	Mouth breathing—daytime and during sleep—is associated with a higher prevalence of AD; risk increased with the greater extent of mouth breathing; this suggests that breathing-related sleep disturbance may contribute to AD pathogenesis

AD and PSO were the most commonly studied conditions, with additional studies on eczema, acne, and CU [6,8,11]. Sleep was assessed using validated tools such as the PSQI [7,9,10], the Children’s Sleep Habits Questionnaire (CSHQ) [12], and the Patient-Reported Outcomes Measurement Information System (PROMIS) sleep scales [1], as well as self-reported questionnaires [2,4]. Large-scale datasets often relied on diagnostic codes [3]. Disease severity was most frequently measured using SCORAD for AD [5] and PASI or DLQI for PSO [7], although some studies used self-rated severity or clinical features [2,11].

Across studies, sleep disturbance affected 30%-70% of patients with dermatologic disease. Greater disease severity, higher itch intensity, and psychological comorbidity were consistently associated with worse sleep [1,4,9]. Pediatric cohorts demonstrated frequent nocturnal awakenings and daytime behavioral consequences [5,11,12], while adults commonly reported reduced productivity and impaired quality of life [4,10]. Genetic evidence supported these findings: insomnia increased the risk of AD and PSO, whereas longer sleep duration reduced the risk of acne and CU [8]. Environmental factors such as nocturnal itching, tobacco smoke exposure, and mouth breathing during sleep further contributed to sleep disruption [6,13].

Quality Assessment

Methodological quality ranged from moderate-high to high (Table 2). Cross-sectional studies scored 15-19 of 20 on the AXIS tool, reflecting clear objectives, appropriate design, valid measurement methods, and transparent statistical reporting. Common limitations included limited sample representativeness, incomplete reporting of recruitment procedures, and insufficient justification of sample size. Large population-based studies more adequately addressed these issues [3,6].

**Table 2:** Summary of the quality appraisal of the included studies Quality assessment of studies investigating dermatologic conditions and sleep outcomes. Cross-sectional studies were appraised using AXIS (Appraisal tool for Cross-Sectional Studies; maximum score 20), case–control studies using the Newcastle–Ottawa Scale (NOS) (maximum 9), and Mendelian randomization studies using Q-Genie (Quality of Genetic Instrument tool; maximum score 77). Scores indicate total points achieved, and quality ratings summarize study reliability. Key methodological notes describe sample representativeness, measurement validity, confounder adjustment, and statistical rigor. Abbreviations: AD – atopic dermatitis; PSQI – Pittsburgh Sleep Quality Index; CSHQ – Children’s Sleep Habits Questionnaire; NHIS – National Health Insurance Service; NHWS – National Health and Wellness Survey; PROMIS – Patient-Reported Outcomes Measurement Information System; UKB – UK Biobank; MR – Mendelian randomization; SCORAD – SCORing Atopic Dermatitis; DLQI – Dermatology Life Quality Index

Study (Author)	Design	Quality Tool	Score	Quality Rating	Key Methodological Notes
Fishbein et al. [1]	Cross-sectional (U.S. PROMIS database)	AXIS	18/20	High	Nationally representative sample; validated sleep tools; no power analysis
Arima et al. [2]	Cross-sectional (NHWS Japan)	AXIS	18/20	High	Propensity-matched population sample; strong analysis; self-reported AD and sleep
Ahn et al. [3]	Cross-sectional (NHIS database, Korea)	AXIS	18/20	High	Large nationwide dataset; robust regression; minor limitation: objective sleep code only (no self-report)
Girolomoni et al. [4]	Cross-sectional (EU5 NHWS)	AXIS	18/20	High	Large multicountry dataset; validated instruments; non-response bias possible
Hamid & Qurtas [5]	Cross-sectional (Erbil, Iraq)	AXIS	16/20	Moderate–High	Valid severity and anthropometric measures; small, single-center sample
Ziyab et al. [6]	Cross-sectional (Kuwait schools)	AXIS	19/20	High	Nationally representative, random sampling; minor reporting limits
Melikoğlu [7]	Case–control (Turkey)	NOS	9/9	High	Rigorous case/control definition, matched sample, validated PSQI, zero attrition
Yang et al. [8]	Mendelian randomization (UK Biobank & FinnGen)	Q-Genie	70/77	High	Strong genetic instrument validity, robust pleiotropy testing, comprehensive sensitivity analysis; large multi-cohort dataset
Sahin et al. [9]	Cross-sectional (Poland)	AXIS	16/20	Moderate–High	Robust stats (mediation model); small sample; single center
Zaky et al. [10]	Cross-sectional (Egypt)	AXIS	17/20	High	Sound analysis; validated Arabic PSQI; single-center sample
Atefi et al. [11]	Cross-sectional (Tehran clinics)	AXIS	16/20	Moderate–High	Valid instruments; limited representativeness; small single-center sample
Kong et al. [12]	Cross-sectional (Korea)	AXIS	16/20	Moderate–High	Validated PSQI/CSHQ; small non-random sample; single-hospital design
Yamaguchi et al. [13]	Cross-sectional (Japan preschools)	AXIS	15/20	Moderate	Large sample but parental self-report; no validated sleep scale

The case-control study achieved the maximum NOS score, indicating strong internal validity [7]. The Mendelian randomization study (Q-Genie appraisal) demonstrated excellent methodological quality with robust genetic instrumentation and comprehensive sensitivity analyses [8]. All studies reported ethical approval and funding sources, supporting overall reliability.

Associations Between Sleep Disturbance and Dermatologic Conditions

Sleep disturbance was highly prevalent across dermatologic conditions, with rates ranging from 16% to over 70%. Poor sleep consistently correlated with greater disease severity, diminished quality of life, and heightened psychological symptoms. Pruritus, pain, and emotional distress frequently mediated these associations, emphasizing the bidirectional relationship between inflammation and sleep dysregulation.

In AD, sleep disturbance was one of the most burdensome symptoms across age groups [1,2,5,11-13]. Increased disease severity was associated with higher rates of sleep impairment, with pruritus emerging as the strongest predictor. Pediatric studies documented additional neurobehavioral consequences, including attention difficulties and hyperactivity [11,12]. Mouth breathing during sleep further contributed to nocturnal arousals in children [13]. Psychological distress - including anxiety and depression - amplified sleep disruption and reduced quality of life [2,4].

In PSO, sleep disturbance ranged from 16% to over 60%, depending on disease severity. Poor sleep correlated with PASI scores, pruritus intensity, and impaired quality of life [7,9,10]. Depression frequently mediated the relationship between disease severity and sleep impairment, underscoring the importance of emotional factors. Younger age, female sex, and higher body mass index (BMI) were additional predictors of poor sleep [9].

Other dermatologic conditions showed similar patterns. Mendelian randomization analyses demonstrated that insomnia increased the risk of AD and PSO, while longer sleep duration reduced the risk of acne and CU [8], supporting a causal bidirectional relationship.

Discussion

This systematic review synthesizes evidence from 13 studies spanning diverse populations and methodological approaches to clarify the association between sleep disturbance and dermatologic disease severity. Across studies, sleep disruption was consistently common, affecting approximately one-third to two-thirds of individuals with skin disease, with higher rates observed in moderate-to-severe conditions. Despite substantial heterogeneity in measurement tools, age groups, and disease categories, a consistent pattern emerged: greater dermatologic disease severity, higher pruritus intensity, and increased psychological burden were strongly linked to poorer sleep outcomes.

AD was the most frequently studied condition, reflecting the well-established burden of nocturnal itching. Findings across pediatric and adult cohorts were largely concordant. Studies using validated severity indices such as SCORAD, POEM, and DLQI demonstrated that worsening AD severity corresponded with increased sleep disturbance, nocturnal awakenings, and impaired restorative sleep [1,5,12]. Pruritus emerged as the strongest and most consistent mediator of sleep impairment. In children, these disturbances extended beyond nighttime symptoms and were associated with measurable daytime behavioral effects, including hyperactivity, attentional difficulties, and reduced school functioning [11,12]. Environmental and physiological modifiers - such as exposure to environmental tobacco smoke, dog-keeping, and mouth breathing - further exacerbated sleep disruption among younger populations [6,13].

In psoriasis, the relationship between disease severity and sleep impairment was similarly evident. Multiple clinical studies demonstrated that PASI and pruritus severity were significantly correlated with poor sleep quality and greater daytime dysfunction [7,9,10]. Sleep disruption in psoriasis may reflect a combination of inflammatory cytokine dysregulation, discomfort from plaques, and psychological distress. Indeed, studies that incorporated mediation models found that depression and reduced quality of life partially explained the association between psoriasis severity and poor sleep [9]. This highlights the multidimensional nature of sleep disturbance, where dermatologic, psychological, and lifestyle components intersect.

Emerging evidence also highlighted disease-specific nuances. Mendelian randomization findings supported a potential causal relationship between insomnia and increased risk of AD and psoriasis, while longer sleep duration appeared protective for acne and chronic urticaria [8]. These genetic data strengthen the plausibility of a bidirectional relationship: skin inflammation disrupts sleep, and sleep dysregulation may in turn exacerbate inflammatory pathways relevant to dermatologic disease. Additionally, non-AD eczema and urticaria cohorts demonstrated that nocturnal itching and sleep fragmentation remain substantial burdens even in the absence of classic AD presentations [3,6].

Despite variability in study design, several unifying themes emerge. First, itch - whether due to AD, psoriasis, or urticarial conditions - remains the most powerful predictor of sleep disturbance. Second, psychological comorbidities such as anxiety, depression, and fatigue frequently co-occur with poor sleep outcomes and may act as both contributors to and consequences of sleep disruption. Third, sleep impairment is associated with broader functional impacts, including reduced productivity, increased healthcare utilization, impaired growth in children, and higher rates of behavioral concerns. The consistent presence of these associations across cultures, age groups, and measurement methods strengthens confidence in the observed relationships.

Overall, this systematic review highlights that sleep disturbance is a core component of dermatologic disease burden rather than an ancillary symptom. These findings emphasize the need for clinicians to routinely assess sleep quality in patients with chronic skin conditions and to integrate sleep-focused interventions - such as itch management, behavioral sleep strategies, or treatment of comorbid psychological symptoms - into comprehensive dermatologic care.

Limitations

This review has several limitations. First, the included studies showed substantial heterogeneity in study design, sleep assessment tools, disease severity measures, age groups, and outcome reporting, which precluded a meta-analysis. Many studies relied on cross-sectional designs, limiting causal inference, and several used self-reported diagnoses or non-validated sleep measures, introducing the risk of misclassification. Sample sizes varied widely, with some studies based on small, single-center populations, reducing generalizability. Although most studies adjusted for key confounders, residual confounding - particularly related to psychological symptoms, socioeconomic factors, and comorbid allergic disease - remains possible. Pediatric and adult data were unevenly represented across conditions, and relatively few studies assessed nighttime physiological parameters (e.g., actigraphy). Finally, publication bias is possible, as studies reporting positive associations may be more likely to be published.

## Conclusions

Sleep disturbance is a significant and multidimensional component of chronic dermatologic disease. Evidence across AD, PSO, CU, acne, and related conditions supports a bidirectional relationship driven by pruritus, pain, psychological factors, and underlying inflammation. These findings underscore the importance of routinely assessing sleep in dermatologic care and adopting multidisciplinary management approaches that incorporate sleep-focused behavioral strategies, psychological support, and targeted anti-inflammatory treatments. Future research should prioritize mechanistic and interventional studies to clarify causal pathways and determine whether improving sleep can enhance both skin-specific and overall health outcomes.
